# Diagnosis of left atrial appendage thrombus in patients with atrial fibrillation: delayed contrast-enhanced cardiac CT

**DOI:** 10.1007/s00330-020-07172-2

**Published:** 2020-09-04

**Authors:** Pietro Spagnolo, Manuela Giglio, Daniela Di Marco, Paola M. Cannaò, Eustachio Agricola, Paolo E. Della Bella, Caterina B. Monti, Francesco Sardanelli

**Affiliations:** 1 Department of Radiology, IRCCS Policlinico San Donato, San Donato Milanese, Milan, Italy; 2 Department of Radiology, Grande Ospedale Metropolitano Niguarda, Milan, Italy; 3 School of Medicine, Vita-Salute San Raffaele University, Milan, Italy; 4 Cardiovascular Imaging Unit, Cardio-Thoracic-Vascular Department, IRCCS San Raffaele Scientific Institute, Milan, Italy; 5 Arrhythmia Unit and Electrophysiology Laboratories, Department of Cardiology and Cardiothoracic Surgery, IRCCS San Raffaele Scientific Institute, Milan, Italy; 6 Department of Biomedical Sciences for Health, Università degli Studi di Milano, Milan, Italy

**Keywords:** Atrial fibrillation, Heart diseases, Thrombosis, X-ray computed tomography, Cardiac imaging techniques

## Abstract

**Objectives:**

The current reference standard for diagnosing LAA thrombi is transesophageal echocardiography (TEE), a semi-invasive technique. We aimed to devise an optimal protocol for cardiac computed tomography (CCT) in diagnosing left atrial appendage (LAA) thrombus in patients with atrial fibrillation (AF), using TEE as reference standard.

**Methods:**

Two hundred sixty consecutive patients referred for radiofrequency ablation for AF were prospectively enrolled. All patients underwent CCT and TEE within 2 hours. The CCT protocol included one standard angiographic phase and three delayed acquisitions at 1-, 3-, and 6-min after contrast injection. Thrombi were defined as persisting defects at 6-min delayed acquisition.

**Results:**

TEE demonstrated spontaneous contrast in 52 (20%) patients and thrombus in 10 (4%). In 63 patients (24%), CCT demonstrated LAA early filling defects at angiographic phase. Among them, 15 (6%) had a persistent defect at 1-min, 12 (5%) at 3-min, and 10 (4%) at 6-min. All 10 thrombi diagnosed on TEE were correctly identified by delayed CCT, without any false positives. For all phases, sensitivity and negative predictive were 100%. Specificity increased from 79% for the angiographic phase to 100% at 6-min. Positive predictive value increased from 16% to 100%. Estimated radiation exposure was 2.08 ± 0.76 mSv (mean ± standard deviation) for the angiographic phase and 0.45 ± 0.23 mSv for each delayed phase.

**Conclusion:**

A CCT protocol adding a 6-min delayed phase to the angiographic phase can be considered optimized for the diagnosis of LAA thrombi, with a low radiation dose.

**Key Points:**

*• In patients with persistent atrial fibrillation referred for ablation procedures, a cardiac CT examination comprising an angiographic-phase acquisition and, in case of filling defects, a 6-min delayed phase may help reduce the need for transesophageal echocardiography.*

*• Cardiac CT would provide morphological and volumetric data, along with the potential to exclude the presence of thrombi in the left atrial appendage.*

## Introduction

Cardiac computed tomography (CCT) is a well-established technique for the evaluation of left atrial and pulmonary vein anatomy [1, 2]. CCT images may be integrated with electrophysiological mapping to guide radiofrequency catheter ablation of atrial fibrillation (AF) [3]. In addition, CCT has been regarded as an emerging noninvasive imaging modality for the detection of left atrial appendage (LAA) thrombus, which is mandatory before radiofrequency catheter ablation for reducing the risk of subsequent thromboembolic events [4, 5].

Although CCT is a highly sensitive modality for excluding intracardiac thrombi, early-phase imaging cannot allow to visually distinguish circulatory stasis from thrombus in the LAA or other cardiac locations, resulting in low specificity and positive predictive value [6–8]. In fact, apparent filling defects on CCT do not always correspond to a thrombus and may represent sludge or severe spontaneous contrast anomalies due to circulatory stasis.

Previous studies have demonstrated an improved diagnostic accuracy of CCT in LAA thrombus diagnosis when an additional delayed acquisition is performed [9, 10]. However, the results are conflicting and the diagnostic accuracy varied widely between reports, preventing the achievement of a definite conclusion and limiting the use of CCT in routine practice in this setting [9, 11, 12]. Moreover, there was a large variability in reported delay times for the late-phase acquisition, ranging from 30 seconds to 3 min, leading to discrepancies between reported specificities and positive predictive values (PPVs).

In this scenario, transesophageal echocardiography (TEE) is considered the standard of care and it is recommended by international guidelines as the only imaging modality for diagnosing LAA thrombus [13, 14], especially in patients with persistent AF, even though it is a semi-invasive test [15, 16].

The purpose of our study was to evaluate the diagnostic performance of delayed CCT scans in diagnosing LAA thrombus in a cohort of consecutive patients with persistent AF referred for radiofrequency ablation, and to determine the optimal delay time acquisition to a differentiate between thrombus and effects of slow-flow state, using TEE as the reference standard.

## Methods

### Study population

Our institutional review board, the Ethics Committee of Ospedale San Raffaele, approved the study (study protocol HSR Late, approved on October 10, 2010), and from December 2010 to March 2013, 260 consecutive patients (199 men (77%), mean age of 59 ± 11 years, range 25–83 years) with drug-refractory, symptomatic, and persistent AF scheduled for radiofrequency catheter ablation using the CARTO (Biosense Webster) or NAVX (St. Jude Medical) system were prospectively enrolled in this study. On the day before radiofrequency catheter ablation, all patients underwent both routine TEE screening and CCT for anatomical evaluation of the left atrium and pulmonary veins, within a 2-hour interval. Exclusion criteria were renal insufficiency (estimated glomerular filtration rate < 30 ml/min), a documented history of anaphylactic reaction to iodinated contrast agent, pregnancy, and the presence of paroxysmal AF. All participating patients provided written informed consent.

All patients were included in an electronic database, all clinical and imaging characteristics were recorded, and the CHADS_2_ score [17] was calculated.

### CCT protocol

All patients were examined with a 64-slice CT scanner (LightSpeed VCT XTe scanner, GE Healthcare). No beta-blockers were used for regulation of heart rate in any of the enrollees. The imaging protocol consisted of a standard angiographic-phase acquisition to evaluate left atrium and pulmonary vein anatomy and three delayed-phase acquisitions at 1-, 3-, and 6-min after contrast material injection, to differentiate thrombus, defined as a persisting defect at 6-min delayed acquisition, from early filling artifacts. The late-phase scanning was performed only if a filling defect (defined as an incomplete visualization or opacification of the entire LAA) was detected in the angiographic phase or if persisted in the 1- or 3-min delayed acquisition.

For the angiographic phase, an electrocardiography (ECG)-assisted scan with prospective gating centered at 75% of the R-R interval was performed within a single breath-hold, covering a range from the aortic arch to the heart base. Tube voltage and current were adapted to patients’ body mass index (100 kVp for patients with a body mass index below or equal to 30 kg/m^2^, and 120 kVp for patients whose body mass index was above 30 kg/m^2^, tube current from 350 to 770 mA), rotation time was 350 ms, and slice thickness was 0.6 mm.

A nonionic, iso-osmolar contrast material (iodixanol, 320 mg of iodine per ml, Visipaque 320; General Electric Healthcare) was administered intravenously at the dose of 80–100 ml, followed by 50 ml of saline solution, at a rate of 5 ml/s by using a dual-shot injector (Nemoto Kyorindo). Visual bolus tracking was performed, and scanning was started when the contrast completely filled the LA.

To minimize unnecessary radiation exposure, delayed scans were limited to the LAA; the 1-min scan was performed only when a LAA filling defect was found at a quick review of the obtained images on the angiographic phase, the 3-min scan when it persisted at 1-min, and the 6-min scan if it persisted at 3-min. All the delayed acquisitions were performed with prospective ECG gating, reduced tube voltage, and current (80–100 kVp, 350–500 mA) and were limited to the LA, in order to further minimize the radiation dose. Particular attention was paid to include the entire LAA in a single slab to avoid possible step artifacts [18]. All CCT images were reconstructed using the adaptive statistical iterative reconstruction (ASIR, General Electric) algorithm (40% filtered back-projection blending) to enable radiation dose reduction without affecting the overall image quality [19].

Images were reconstructed using a soft-tissue convolution kernel.

The scanning length and time as well as the dose length product (DLP) of the CCT were noted from the scanner console, and the effective radiation dose of the different phases was calculated by multiplying the DLP times a conversion factor (0.014 mSv/cGy/cm), as previously suggested [20].

### Image analysis

Images were transferred to an external workstation (Aquarius Intuition; TeraRecon). Two independent radiologists with 14 and 9 years of experience in cardiovascular imaging, blinded to all patients’ data, qualitatively evaluated the presence of LAA filling defects on both angiographic and delayed scans. The persistence of a LAA filling defect on the last 6-min delayed CCT phase was considered as indicative of the presence of LAA thrombus whereas a disappearance of the filling defect at 1-, 3-, or 6-min suggested the presence of slow-flow effect and pseudo-filling defects. Differences in the assessment by the readers were resolved by consensus.

For each patient, anatomy of the left atrium and the pulmonary veins and left atrium volume were evaluated in the angiographic-phase images. On the day of pulmonary vein isolation, 3D CCT images were reconstructed on a separate workstation and integrated with electro-anatomical mapping.

### Transesophageal echocardiography

All patients underwent TEE examination using a GE Vivid E9 (GE Healthcare) ultrasound system equipped with 6VT-D probes, respectively. A complete 2D, colored, pulsed, and continuous-wave Doppler echocardiogram was performed according to EACVI recommendations [13]. The exams were digitally stored and transferred on external workstation for offline analysis (EchoPAC, version 201).

TEE allows the following information: (1) a 2D multiplane or 3D analysis of LA and LAA anatomy, (2) a semi-quantitative classification of the degree of spontaneous echo contrast, and (3) a Doppler measurement of the LAA emptying flows.

Spontaneous echocardiographic contrast was defined as an intracavitary swirling smoke-like echo within the left atrium or LAA that could not be eliminated by altering the gain settings, and it was classified as mild to moderate, severe, and sludge (dense smoke, viscid echodensity, not solid) [21]. LAA thrombus was defined as a solid, well-circumscribed mass that was visible throughout the cardiac cycle [22].

Two experienced cardiologists blinded to CCT and clinical data reviewed all TEE images. Differences in assessments by the observers were resolved by consensus.

A thrombus was defined as a circumscribed echogenic or echolucent mass distinct from the surrounding atrial wall. Spontaneous echocardiographic contrast was defined as an intracavitary swirling smoke-like echo within the left atrium or LAA that could not be eliminated by altering the gain settings. Spontaneous echocardiographic contrast severity was classified using a 4-grade scale based on appearance and density (none, mild, moderate, or severe), as previously described [23, 24].

### Statistical analysis

All statistical analyses were performed using SPSS, version 17.0 (SPSS, Inc.). Normally or near-normally distributed continuous variables were expressed as mean ± standard deviation and categorical variables as frequencies or percentages. Ninety-five percent confidence intervals (CI) were calculated according the binomial distribution.

Using TEE as the reference standard, the diagnostic performance for diagnosing LAA thrombus of early- and delayed-phase CCTs was calculated from contingency tables. DLP (mGy × cm) of all examinations was recorded, and the effective radiation exposures, estimated in mSv, were compared between patients with and without thrombus or circulatory stasis by Student’s *t* test for independent samples. Inter-reader reproducibility was appraised with Cohen’s *κ* and calculating raw concordance. Cohen’s *κ* was interpreted according to guidelines by Koo and Li [25]. A *p* value < 0.05 was considered statistically significant.

## Results

The clinical characteristics of the overall study population are summarized in Table 1. A total of 260 consecutive patients were enrolled in the study, and no enrolled patient was excluded according to exclusion criteria. Out of all patients, 119 (46%) had a CHADS_2_ score of 0, 124 (48%) had a CHADS_2_ score of 1, and 17 (6%) had a CHADS_2_ score ≥ 2. All patients underwent CCT and TEE without complications. All patients were in AF during CCT images acquisition. The average heart rate was 86 ± 25 (range 66–114). In all cases, image quality was technically adequate for clinical assessment.

**Table 1:** Baseline characteristics of the study population

Variable	Values (*n* = 260)
Age (years), mean ± SD (range)	59 ± 11 (25–83)
Males, *n* (%)	199 (77)
Body mass index (kg/m^2^), mean ± SD (range)	27.2 ± 5.1 (17.8–49.1)
Hypertension, *n* (%)	133 (51)
Diabetes mellitus, *n* (%)	16 (6)
Hyperlipidemia, *n* (%)	71 (27)
Ejection fraction ≤ 55%, *n* (%)	21 (8)
Previous TIA/stroke, *n* (%)	7 (3)
Mean CHADS_2_, mean ± SD (range)	0.6 ± 0.6 (0–3)

*TIA* transient ischemic attack; *CHADS*_*2*_ Congestive Heart Failure, Hypertension, Age > 75 years, Diabetes and Prior Stroke

Of 260 patients, 10 (4%, 95% CI 2–7%) were diagnosed with thrombi and 52 (20%, 95% CI 15–25%) with spontaneous echocardiographic contrast (35 mild, 14 moderate, and 3 severe) without thrombus on TEE. All thrombi were located in the LAA.

**Fig. 1:**
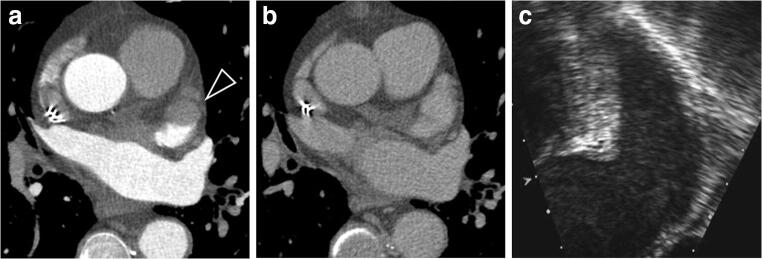
Axial cardiac images of a 70-year-old man. **a** Early-phase CCT image demonstrated a filling defect in LAA (black arrowhead). **b** CCT image 1-min after contrast injection demonstrated no filling defects in LAA. No further acquisitions were needed. **c** TEE image obtained 2 hours after CCT demonstrated moderate spontaneous echographic contrast with no thrombus in LAA

**Fig. 2:**
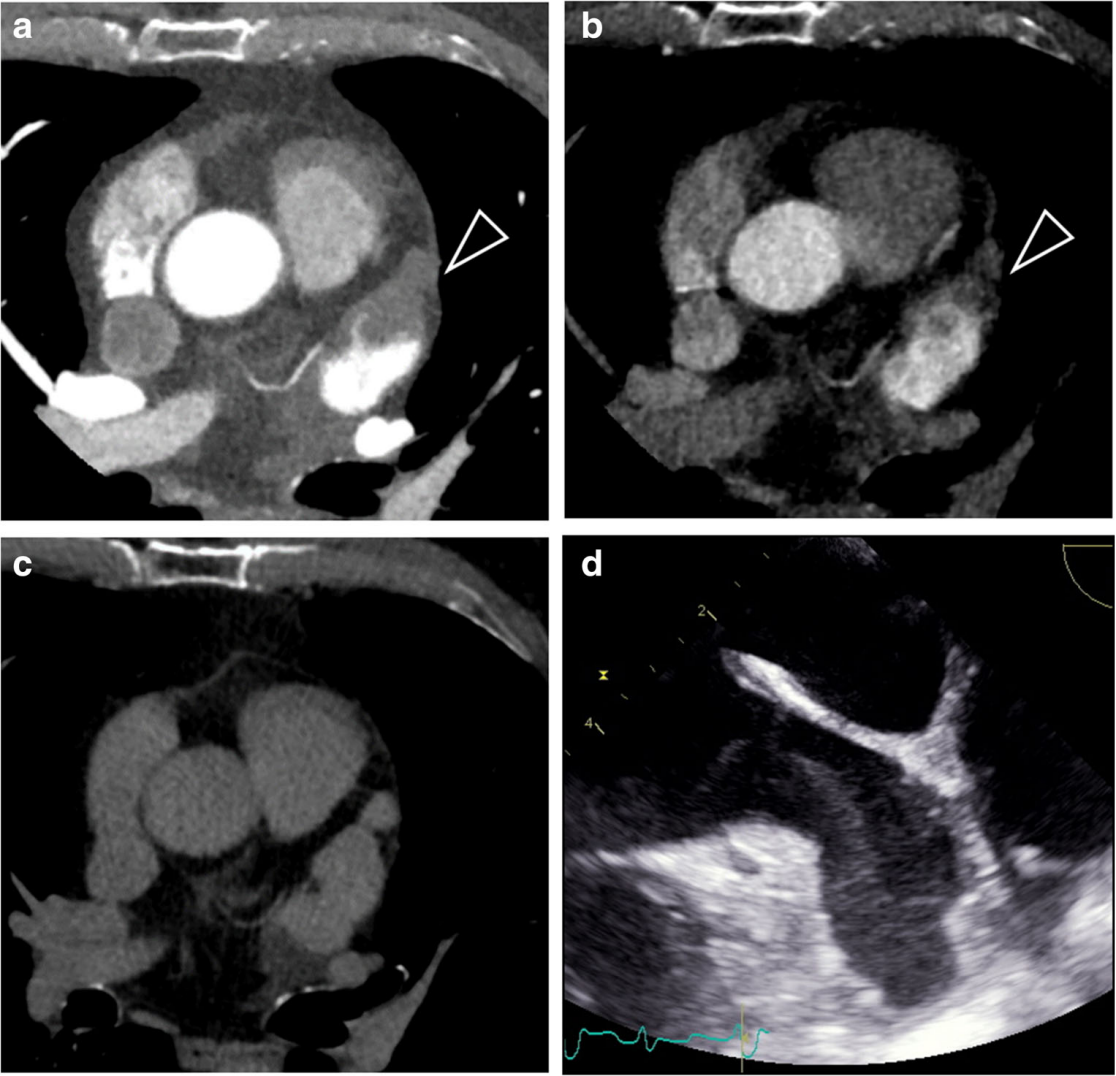
Axial cardiac images of a 58-year-old woman. **a** Early-phase CCT image demonstrated a filling defect in LAA (black arrowhead), persisting in the late-phase CCT image 1 min after contrast injection (**b**). **c** The following scan at 3-min did not confirm the filling defect. **d** TEE image obtained 2 hours after CCT image acquisition demonstrated severe spontaneous echographic contrast with no thrombus in LAA

**Fig. 3:**
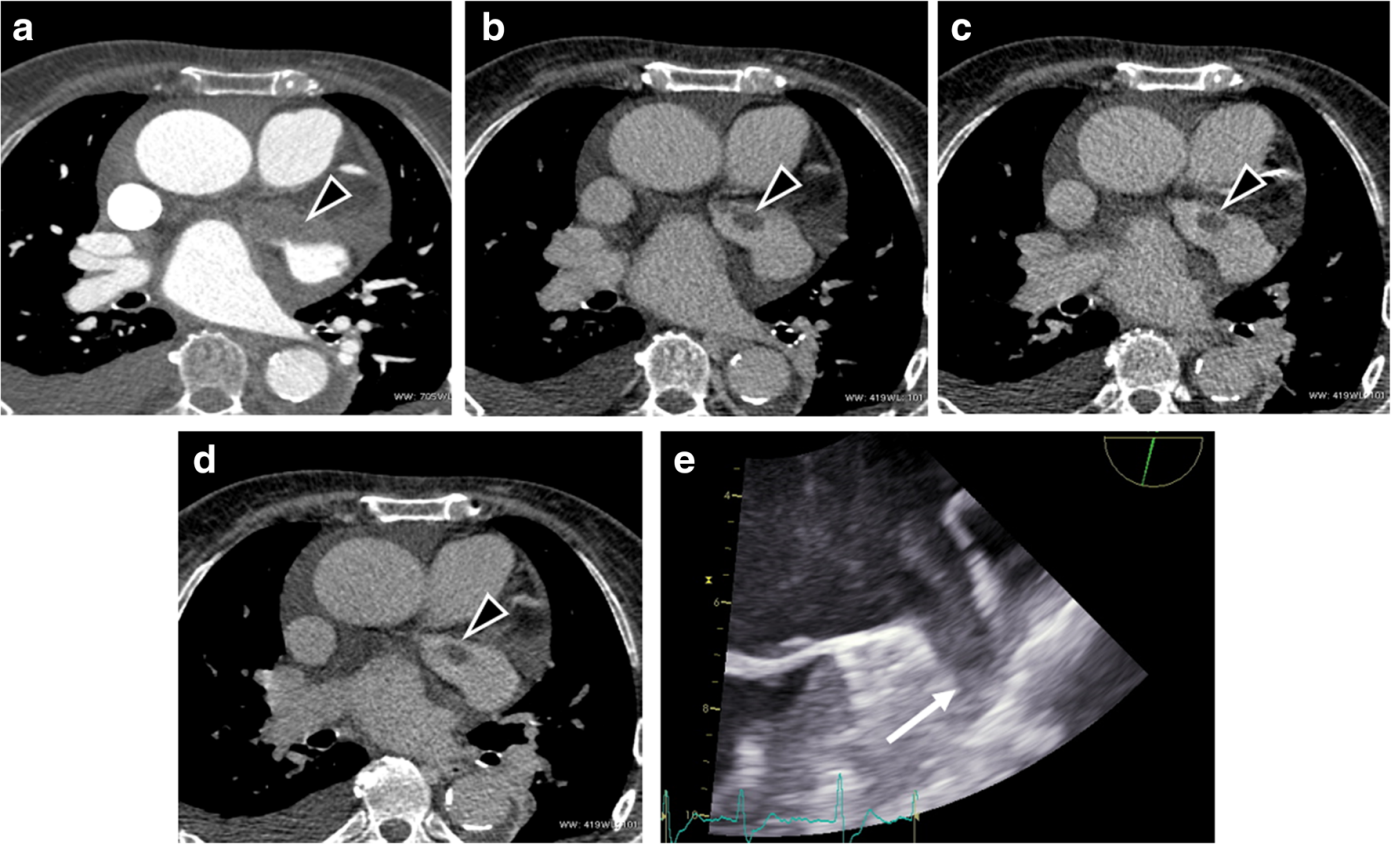
Axial cardiac images of a 53-year-old man. **a** Early-phase CCT image demonstrated a large filling defect in LAA (black arrowhead) with a small persisting quote in both the multiple late-phase scans performed (**b**–**d**), suggestive of thrombus in LAA. **e** TEE image obtained 1 h after CCT confirmed LAA thrombosis (white arrow)

As depicted on the CCT, 63/260 patients (24%) presented with LAA early filling defects on the angiographic acquisition. Of these, 15/260 (6%) had a LAA persistent filling defect at 1-min and underwent 3-min delayed acquisition. Then, 12/260 (5%) patients still had a pseudo-filling defects at 3-min, so the 6-min delayed acquisition was performed. The remaining 10/260 (4%) filling defects were persistent on the last 6-min delayed scan and were diagnosed as thrombus (Figs. 1, 2, and 3).

Interobserver agreement was excellent (*κ* = 0.950, *p* < 0.001), and so was raw concordance (99.7%). In one case, there was disagreement between the two readers; in that case, consensus was achieved at a joint reading and final diagnosis was no filling defects.

TEE and CCT results are summarized in Table 2.

**Table 2:** Concordance between delayed-contrast cardiac computed tomography and transesophageal echocardiography for the detection of left atrial appendage thrombus

CCT	TEE (*n* = 260), *n* (%)	
	Thrombus	No thrombus
Angiographic phase		
Filling defect	10 (4)	53 (20)
No filling defect	0	197 (76)
1-min delay		
Filling defect	10 (4)	5 (2)
No filling defect	0	245 (94)
*p* value with previous timing < 0.001		
3-min delay		
Filling defect	10 (4)	2 (1)
No filling defect	0	248 (95)
*p* value with previous timing 0.437		
6-min delay		
Filling defect	10 (4)	0
No filling defect	0	250 (96)

*p* value with previous timing 0.663

**Fig. 4:**
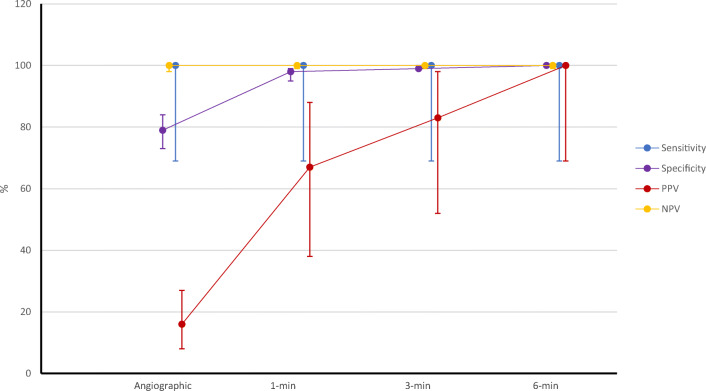
Diagnostic performance of different CCT phases, expressed as sensibility, specificity, positive predictive value (PPV), and negative predictive value (NPV)

Sensitivity, specificity, PPV, and negative predictive value (NPV) of CCT at all phases using TEE as a reference standard are reported in Table 3, while Fig. 4 depicts their changes.

**Table 3:** Diagnostic accuracy parameters for scans acquired at different timings post contrast injection

	Sensitivity	Specificity	Positive predictive value	Negative predictive value
Angiography	100 (69–100)	79 (73–84)	16 (8–27)	100 (98–100)
1-min delay	100 (69–100)	98 (95–99)	67 (38–88)	100 (99–100)
3-min delay	100 (69–100)	99 (97–100)	83 (52–98)	100 (99–100)
6-min delay	100 (69–100)	100 (99–100)	100 (69–100)	100 (99–100)

Data are reported as value (%) and 95% confidence interval

The estimated radiation dose was 2.08 ± 0.76 mSv (1.19–5.11 mSv) for angiographic imaging and 0.45 ± 0.23 mSv (0.18–1.08 mSv) for every single late-phase scan. Considering all patients, the mean radiation dose of the complete CT examination was 2.17 ± 0.91 mSv (1.19–5.71 mSv), while the maximum radiation dose estimated in patients who underwent all delayed-phase scans (*n* = 12, 5%) was 3.29 ± 1.05 mSv (1.90–5.71 mSv). The total radiation exposure was significantly higher for patients with thrombus (3.29 ± 1.05 mSv) compared with both patients without early filling defects (2.08 ± 0.76 mSv) and with circulatory stasis (2.29 ± 0.92 mSv) (*p* < 0.001 and *p* = 0.003, respectively). Conversely, no significant differences in radiation exposure were noted between patients with circulatory stasis and without early filling defects (*p* = 0.080).

## Discussion

Over the last years, the role of CCT in assessing LAA thrombus has been supported by a growing number of reports, showing that it is a potentially useful imaging modality, distinctly when delayed-imaging acquisition protocols are used [24, 26, 27].

A meta-analysis published by Romero et al [9] demonstrated that the use of a two-phase strategy, including delayed imaging, significantly improves CCT diagnostic specificity, leading to a pooled PPV of 92% compared with a PPV of 41% for the angiographic phase, while the NPV remains excellent (approximately 100%). These results are consistent with the more recent retrospective study by Lazoura et al [28] who obtained both a specificity and a PPV of 100% by experimenting a 60-s delayed CCT acquisition in 122 patients undergoing left atrial intervention for AF, using TEE as reference standard. Hence, the addition of a delayed acquisition strongly reduced CCT false positive rates since a longer time is available for the contrast media to fill the entire LAA, thereby yielding greater contrast opacification and improving differentiation of thrombus from circulatory stasis.

Despite the promising diagnostic performance of the two-phase CCT protocol, there were no data from human or animal studies about the basis of a correct scan timing for the late-phase acquisition. Variable delay times were reported, ranging from 30 s to 2 or 3 min, leading to discrepancies between reported specificities or PPVs, particularly in patients who were in AF at the time of the study.

Moreover, the suboptimal diagnostic performance of CCT in this setting is partly due to a high heterogeneity of populations (heterogeneous cohorts of patients with different risk profiles and prevalence of thrombosis), different time intervals between CCT and TEE, and no clear cutoff value for CCT quantitative analysis [9, 12]. As a consequence, to date, CCT has not been included in practice guidelines for evaluation of patients with AF, even if this modality is performed before AF ablation for anatomical left atrium characterization and could be implemented to diagnose LAA thrombus before the procedure. Thus, a standardization of the delayed-phase CCT protocol is desirable.

In this clinical experience, we found that even 3-min delay for dual-phase CCT is not always appropriate to differentiate thrombus from circulatory stasis, still leading to false positive cases. We hypothesize that this might result from the slow-flow artifacts related to the physio-pathological aspects of persistent AF and LAA dysfunction. In fact, contrast opacification may take longer in these patients. For these reasons, we studied a selected population of patients with persistent AF. On the other hand, we prolonged the delay of the late-phase scan to completely eliminate filling defects related to slow-flow states, while maintaining a vascular attenuation high enough to allow thrombus detection.

Therefore, our study was designed to derive the optimal delay time for use in real-world clinical practice. Differently from other investigations, our data were obtained in a large and homogeneous population of candidates to radiofrequency catheter ablation, all with persistent AF. Patients with paroxysmal AF were excluded from our study because of their demonstrated very low incidence of both pseudo-filling defects and LAA thrombosis, making TEE unnecessary before a planned AF ablation [29]. To improve the robustness of the study, we minimized the interval between TEE, CCT, and pulmonary vein isolation through close collaboration with cardiologists.

Our main findings are that 5 persistent filling defects (33%) at 1-min delay scan and 2 (17%) at 3-min delay scan, respectively, disappeared at 6-min delayed acquisition and would have been falsely diagnosed as LAA thrombus, resulting in suboptimal PPVs of 67 and 83%, respectively.

Considering the 6-min delayed acquisition, both sensitivity and PPV increased to 100%, while maintaining an optimal delineation of thrombus dimensions and morphology, fully comparable with TEE results. Other options for technical adjustments to improve LAA thrombus detection described in previous studies include a biphasic iodine bolus technique [30]. However, while this method has the advantage of allowing for shorter scan times, it still presents a higher rate of false positives compared to the 6-min delayed acquisition.

Similarly to other investigations, our study reaffirms that the prevalence of LAA thrombosis in candidates to pulmonary vein isolation is low (4%), as the majority of these patients are anticoagulated and have a low prevalence of CHADS_2_ score ≥ 2 (6%). Our large sample size (260 patients) makes this estimation relatively precise (95% CI 2–7%)

Our study has some limitations. First, the use of additional late-phase acquisitions caused an increased radiation exposure. However, we minimized radiation burden by using body mass index–adapted current and voltage modulation and prospective ECG triggering for both angiographic and delayed scans. In addition, unenhanced scan and calcium scoring were not performed and the delayed scans were performed only in a small number of patients and limited to the left atrium (delivering only 0.45 ± 0.23 mSv additional dose). The resultant effective radiation burden was thus acceptable and in line with recent publications. In clinical practice, using only the angiographic acquisition and the 6-min scan limited to the LA, full diagnostic information can be obtained with a low radiation exposure. Second, we considered TEE as a reference standard even though it is heavily operator dependent and has suboptimal diagnostic accuracy in LAA assessment. Because of the complex anatomic features of thrombi, they can be difficult to detect and thus missed while a false positive diagnosis can result from misinterpretation of heavy spontaneous echo contrast or pectinate muscles as thrombi. Hence, even though TEE is the standard of care in this setting, future prospective studies of CCT using surgical or pathologic reference standard are desirable. Moreover, our selection of CCT delay times (1-, 3-, and 6-min) was arbitrary, based on our previous clinical experience. The optimal delay time can vary widely according to different factors, as well the severity of left atrium and LAA dysfunction and the contrast injection protocol (intravenous injection rate, total injection volume, saline flush). Another limitation of our study is the fact that we did not include a quantitative analysis of CCT scans, and only a qualitative assessment was performed. This was due to this work, a preliminary, visual analysis that could be easily integrated into clinical practice of findings stemming from delayed phases concerning LAA thrombus detection. Further studies including quantitative analyses and possibly aiming to contrast agent dose reduction and optimization will be performed [31].

In conclusion, we showed that CCT protocol including delayed phases is highly accurate for diagnosing LAA thrombosis and may obviate TEE in patients undergoing CCT for ablation procedures. Because the 1- and 3-min phases are still influenced by circulatory stasis leading to false positives, adding a 6-min delayed phase to the angiographic phase can be considered an optimized CCT protocol in this setting, with an overall radiation dose lower than 3 mSv. A dual (early and 6-min) phase CCT might represent the modality of choice to exclude LAA thrombus before AF catheter ablation. Given the very low prevalence of LAA thrombus, the vast majority of patients will have a negative test, waiving the need for routine TEE. Future multicenter studies are needed to prove the effectiveness of this approach.

## Abbreviations

AF: Atrial fibrillation
CCT: Cardiac computed tomography
DLP: Dose length product
ECG: Electrocardiography
LA: Left atrium
LAA: Left atrial appendage
NPV: Negative predictive value
PPV: Positive predictive value
TEE: Transesophageal echocardiography
